# What Influences Patient Participation in an Online Forum for Weight Loss Surgery? A Qualitative Case Study

**DOI:** 10.2196/ijmr.2847

**Published:** 2014-02-06

**Authors:** Anita Das, Arild Faxvaag

**Affiliations:** ^1^Department of NeuroscienceFaculty of MedicineNorwegian University of Science and TechnologyTrondheimNorway

**Keywords:** obesity, eHealth, bariatric surgery, online forum, communication, social support

## Abstract

**Background:**

Many patients who undergo weight loss (bariatric) surgery seek information and social support in online discussion forums, but the vast amount of available information raises concerns about the impact of such information. A secure online discussion forum was developed and offered to bariatric surgery patients. The forum was moderated and allowed contact with peers and health care professionals.

**Objective:**

The purposes of this study were to explore how individuals undergoing bariatric surgery used the moderated discussion forum and to better understand what influenced their participation in the forum.

**Methods:**

The study was designed as an explorative case study. We conducted participant observation of the discussion forum over a time period of approximately six months. For further insight, we carried out in-depth semistructured interviews with seven patients who had access to the forum. We analyzed the material inductively, using content and thematic analysis.

**Results:**

The patients used the forum as an arena in which to interact with peers and providers, as well as to provide and achieve informational and social support. The analysis suggests that there are three major themes that influenced participation in the online discussion forum: (1) the participant’s motivation to seek information, advice, and guidance, (2) the need for social support and networking among peers, and (3) concerns regarding self-disclosure.

**Conclusions:**

The findings of this study imply that a moderated discussion forum for bariatric surgery patients has potential for use in a therapeutic context. The discussion forum fulfilled the informational and support needs of the bariatric surgery patients and was particularly useful for those who excluded themselves from the traditional program and experienced barriers to expressing their own needs. Even though our findings imply that the patients benefitted from using the forum regardless of their active or passive participation, restraining factors, such as considerations regarding self-disclosure, must be further investigated to prevent certain users from being precluded from participation.

## Introduction

### Bariatric Surgery Patients

The number of people suffering from obesity has risen globally in the last decade, and comorbidities such as metabolic syndromes, respiratory problems, coronary heart disease, cancer, and psychosocial challenges are all closely associated with obesity [1-3]. Weight reduction has beneficial health effects on obesity-related comorbidities and mortality, and the demand for weight loss interventions has therefore risen [4,5]. Weight loss can be achieved through lifestyle interventions, pharmacotherapy, and/or surgery, but a number of people do not achieve the desired weight reduction [6,7]. Bariatric surgery has been shown to be the most effective intervention and to produce significant initial weight reduction in the great majority of patients, but it is mainly reserved for the severely obese who fail to lose weight through conventional methods [8]. The purpose of the surgery is to restrict food intake, but it also contributes to reduced absorption, which leads to poor digestion and the reduced uptake of several nutrients. Thus, patients must take lifelong vitamin supplements [9,10]. Also, the surgery requires patients to undergo substantial lifestyle changes, including adjustments to eating behavior and physical activity. However, noncompliance with the postsurgery recommendations is pervasive, and a number of patients regain weight and experience nutritional deficiencies after some time has elapsed [11-17]. Providing support for bariatric surgery patients is an essential part of the treatment program because weight regain, nutritional problems, and metabolic problems can be prevented or treated [16,18].

### Online Support Forums

The Internet has become an important health care medium, giving people the opportunity to search for information, guidance, and social support. Online health resources are particularly relevant for patients who may encounter barriers to obtaining information on self-management and coping strategies [19,20]. In general, self-management activities are associated with successful long-term weight maintenance [14,21-25], and studies imply that social support may encourage compliance with postsurgery recommendations [26-28]. Studies on other patient groups show that online social support may include benefits such as enhanced health literacy, improved quality of life, and patient empowerment [29-32]. Some patients achieve considerable social support in their real-life environments, but a number of patients also participate in health-related forums on the Internet. Being aware of the lack of social support that some patients experience is important in providing complete health care service for these patients. Using health-related online forums has been shown to have an overall positive effect on the degree to which people are able to cope with the situations they are facing, both socially and as regards their conditions [20]. Hwang et al suggest that by addressing diet, physical activity, and motivation in a comprehensive approach, one can meet the needs of obese patients after surgery [33]. Using online forums to address these issues has the potential to support this patient group.

Most health-related online forums are dominated by peer-to-peer communication, without professional supervision or involvement [20]. In these forums, the quality and credibility of the available health information is mixed, which raises concerns about their impact and value [34]. Eysenbach et al reviewed publications on the effect of online peer support groups, but could not find any isolated outcomes of the peer support groups controlling for other interventions [35]. Research shows that patients want professionals to take an active role in such forums [36,37], and some studies indicate that facilitated or moderated communities are more beneficial [19,38]. Lindsay et al found that having a moderator in an online support group influences compliance in terms of maintaining healthy behaviors and reducing health care visits [19]. Klemm identified that the participants in moderated online support groups for breast cancer patients read and posted significantly more than in peer-led groups [39]. Ryan performed a study on trust and participation in two online self-help communities, one moderated and one unmoderated, and his primary finding was that the moderation process prevented any communication from disruptive individuals [38]. The unmoderated community challenged disruptive and suspicious individuals, resulting in hostile discussions, while the moderated community encouraged social communication, experienced more participation, and facilitated the accumulation of a history-based trust [38].

We here report from a case study exploring how bariatric surgery patients used a moderated discussion forum in the context of bariatric surgery treatment. It is further intended to address the factors that influence their participation. By identifying these aspects, we aim to gain an improved knowledge of how such a solution can be used as part of a bariatric surgery program.

## Methods

### Study Setting

The online discussion forum under study was one of many features of a secure eHealth portal. The eHealth portal was developed for patients undergoing bariatric surgery and included health-related information, self-management tools, and communication features [37,40]. The portal was developed through a human-centered design process [41], and according to the security and privacy concerns that are required for such solutions in Norway [37,40]. To gain access to the portal, the user was required to be registered in the system and obtain a username and password. For authentication purposes, the user would receive a one-time pin code via text message that he or she would then enter during the log-on process.

The communication features of the portal included an online discussion forum and personal one-to-one communication (patient-to-patient and health care professional-to-patient or vice-versa). Posting on the forum required that the users appeared with their real names, which was necessary in order for it to be used in a medical context. One person from the research team had the role of moderator of the forum and could monitor the discussions and take action if inappropriate messages were posted, which was one of the requirements that was identified during the human-centered design process. The moderator was educated in nursing and could comment on the postings that were within her field of competence. She also had the responsibility of posting weekly topics that were relevant to the patient group. These topics were either initiated by the clinic or created after requests from the patients. There were five health care professionals (one psychiatric nurse, one head nurse, two nurses, and one dietician) at the clinic that had access to the eHealth portal and had the responsibility of facilitating the patients through the portal and answering their requests. Further, these five professionals could make contact with other professionals for additional counseling if necessary.

### Participant Inclusion

This study was designed as an explorative case study. The selection criteria for patient inclusion were as follows–18 years or older, basic proficiency in the Norwegian language, and enrollment in a bariatric weight loss program at the hospital. Participants provided written consent when enrolling in the study. The study followed the guidelines of the Declaration of Helsinki and was approved by the regional Ethics Committee (Trondheim, Central Norway) and by the Norwegian Social Science Data Services. Participants were recruited at the bariatric surgery clinic, where the first author made contact with potential participants, provided information about the study, and invited them to participate. The inclusion period lasted for one month, from the middle of May to the middle of June of 2011. Initially, 65 patients were asked to participate. There were 60 patients that agreed and obtained access from the time of recruitment until the middle of December of 2011. Demographic data were collected through questionnaires developed for this study (Tables 1 and 2).

**Table 1:** Demographic data of the patients who had access to the discussion forum through the eHealth portal.

Characteristic		Total n available	n (%) or mean (SD)
Age, mean (SD)		60	40 (SD 9.3)
Female, n (%)		60	45 (75)
**Highest education completed, n (%)**		57	
	Primary school		4 (7)
	High school		30 (53)
	University/College		23 (40)
**Employment status, n (%)**		60	
	Full/part time work		40 (66)
	Student		3 (5)
	On sick leave		4 (7)
	Unable to work/disabled		9 (15)
	Unemployed		3 (5)
	Other		1 (2)
Have undergone surgery, n (%)		60	56 (94)

**Table 2:** Demographic data, interview participants.

Informant	Age	Gender	Highest education completed	Time of surgery
Anne	30-35	Female	University/College	Spring 2011
Kari	50-55	Female	University/College	Winter 2010/2011
Frank	45-49	Male	High school	Spring 2011
Linn	25-29	Female	High school	Waiting to undergo
Monica	30-35	Female	Primary school	Summer 2010
Nina	40-45	Female	High school	Spring 2011
Kristin	30-35	Female	High school	Autumn 2010

### Discussion Forum

Via the eHealth portal, the patients had access to the online discussion forum. We conducted participant observation of the forum during the access period, and when the period ended, we retrieved all postings to the online forum and analyzed the posts inductively using qualitative content analysis [42]. The analysis was performed in a stepwise process in which both authors reviewed and coded the transcripts individually before the findings were compared and refined in a consensus decision-making process. We used English terms and concepts during the analysis and used HyperResearch software to facilitate the process. The extracts from the discussion forum that are reported in this paper were translated from Norwegian into English by the first author before the second author reviewed the translation.

### Interviews

To obtain a better understanding of the users’ activities in the discussion forum, we conducted interviews with users who had access to the portal. Informants were recruited through the discussion forum, where the first author posted an invitation to take part in interviews. A stratified purposeful sampling was made in terms of the variables age, gender, and time of surgery in order to ensure variation among the participants. There were 8 patients that agreed to interviews, but one failed to show up. We carried out semistructured, in-depth interviews with seven informants at the university research center between September and December of 2011. The interviews were conducted in Norwegian and lasted between 44-108 minutes, having a typical duration of 60 minutes. We used open-ended questions, for example, “How is your daily life (if operated on, after surgery)?” “What are your experiences with using the discussion forum?” “How do you experience the fact that your real name appears when you post to the forum?” “What are your feelings about the lack of anonymity?” The semistructured form of the interviews allowed the researcher to include questions related to emerging themes during the interview. All interviews were sound recorded and transcribed verbatim before analysis. When the last two interviews were analyzed, we did not identify new emerging themes and decided that we had reached saturation. HyperResearch software was used to facilitate the process of analysis, which was done inductively by using thematic analysis [43]. Both authors reviewed the interviews and analyzed the data. In the first stage of the thematic analysis, both authors made themselves familiar with the data and read through all the transcripts before they created initial codes of the data individually. In the next stage of the process, the codes were collated, and concepts were generated. These were then compared, contrasted, and discussed in light of the relevant literature and theory, and the final themes were achieved via consensus. This was done to ensure that our findings were coherent and increase the validity of the findings. The interview transcripts were in Norwegian, but the process of analysis was performed in English, using English codes and concepts. The quotes in this paper were translated from Norwegian into English by the first author before the second author reviewed the translation. The names reported in this paper are pseudonyms and not the real names of the participants.

## Results

### The Three Themes

Through the analysis, we identified three major themes that influenced participation in the online discussion forum: (1) the participant’s motivation to seek information, advice, and guidance; (2) the need for social support and networking among peers; and (3) concerns regarding self-disclosure.

### Informational Support, Guidance, and Advice

By observing the discussion forum, we identified the fact that the patients used the forum as an arena in which to provide and obtain informational support. Patients that undergo bariatric surgery must perform a number of self-management activities in order to achieve and maintain weight loss. Also, they must adjust their dietary habits to avoid malnutrition and other negative repercussions of the surgery. The informants who had undergone surgery mentioned that these considerations made them feel insecure. Therefore, they began to search for information and guidance regarding how to manage their *“*new lives.” Frank had undergone surgery six months before the interview and found himself continually searching for information.

You are afraid about what you can eat. It says that you should be aware of rice and such, but you haven’t got any information about whether you can to eat it now, after so long a time. You don’t know anything about that. It is the first phase that is [described]…and then you have to try things yourself.

Frank

Insecurity related to coping with their new lives was a recurring topic, and the possibility of contacting health care professionals through the forum was highly appreciated by all the informants. Kristin remarked that she found it “brilliant*”* to have this opportunity. Anne described the professional guidance one could obtain as *“*the advantage of this forum as compared to the other ones.” Forum observations revealed that some patients approached the health care professionals directly with specific questions, for example, *“*How often are we supposed to take blood tests at our primary care doctor to determine whether we are taking the correct dose of vitamins?” Others simply reported their general experiences, for example, *“*When the weather is hot, I experience dumping (repercussion of the operation, experienced as uneasiness) more quickly, and it is caused by foods and drinks that I normally tolerate,” to which the professionals could then respond. The moderation process involved health care workers understanding patient challenges and taking actions accordingly, such as assigning the patient to a regular consultation for further investigation if necessary. We observed that in some cases the patients required informational and instrumental support, while in other instances the needs were of a more emotional or social character. Every week the moderator published a relevant topic on the discussion forum, and the participants would receive a reminder about it on email. The topics related to food, diet, nutrition, exercise, and practical information. We observed that these postings triggered further comments and questions from the patients, and those interviewed commented that these weekly topics motivated them to continue using the forum–“I like that I get that email about the weekly topic because then, I get a reminder to go in” (Kristin).

Some informants reported that they experienced difficulties in making direct contact with the professionals due to personal barriers. When they began using the forum they discovered the benefit of connecting with professionals via the forum, rather than waiting for an appointment or making contact via phone, as Monica expressed.

I think it is very positive that you can ask questions that are conveyed to a dietician or a doctor because I must admit that picking up the phone and asking someone is very challenging. That barrier–I think it is difficult. What if it’s only me? How ridiculous! You get that feeling. Then, it is easier to write online.

Monica

This was supported by the other informants, and the convenience of the asynchronous aspects of such communications were also seen to be beneficial–“It’s easier to go in here, ask questions, and get answers, rather than calling around and stuff” (Anne). Kristin underlined the advantage of connecting with both peers and professionals through the forum.

The fact that you have others who have gone through it themselves to talk to and that you can ask health care workers about things you wonder about makes participating in the forum easier than persuading oneself to make a phone call…so this is good…one has complete health care service.

Kristin

Thus, the possibility of making contact with both professionals and peers through the same forum was an advantage that they had not experienced before.

### Social Support and Networking Among Peers

Some patients experienced the first period after surgery as particularly difficult because it was characterized by uncertainty and a lack of information.

It is undoubtedly the first period, the first three weeks [after surgery], when you have the most questions. Can I eat this? What can I do now? Because clearly, it is mentally tough too.

Kari

The emotional and psychological factors related to surgery were recurring topics among the patients, and the need for social and emotional support was clear. The online forum became an arena in which they could introduce and discuss sensitive matters that they would not have discussed in another setting. As Anne said, “I think it is easier to talk about them (sensitive issues) in a place like this than face-to-face.” Kristin remarked that one could have different attributes online than in real life, enabling the discussion of problems that one otherwise would have kept to oneself.

You can be much tougher on the Net, write things that you might not want to say to people because they are difficult to talk about. This becomes easier when you have a screen you can hide behind.

Kristin

Many of them experienced challenges in their daily lives related to the weight loss treatment, many of which were of a motivational or psychosocial character. In some cases, the value of peer support and understanding was extremely important in order to maintain inner motivation, as Linn revealed.

So you go into a downturn just by talking to a person that doesn’t know what you are talking about. Then, it is more important to talk to a person who has been there, who knows what you have been through, who can encourage you to continue.

Linn

Sharing personal stories and narratives was an important part of the forum, and the topics covered related to the challenges of losing weight, motivational difficulties, and the everyday experiences of the patients. When asked about the motivation behind this, they reported that the aim was to promote acknowledgement, emotional support, and approval. Anne explained this as follows–“It is actually the support and the approval regarding what you are doing, feedback regarding whether it is right, and feedback regarding insecurities.” A few patients created “threads” in the discussion forum that they named *“*diaries,” where they wrote their personal diary notes with details about their daily life experiences and challenges. The following excerpt is from the initial post written by one of the diary writers.

I think it is more enjoyable to write a “diary” that everyone can read and comment on. I like to get feedback on how I do things, what I eat, and thoughts that I have about the surgery and about life after the operation, so here comes a little of everything…Hope you will read and comment.

Diary writer

The excerpt shows that the diary writer was aware and honest about her own intentions to share her personal experiences from the beginning. We observed that the diary writers received feedback and comments on their writings from other patients, as well as from the health care professionals. The replies were often of a supportive and motivational nature and were regularly offered when the narrator expressed the need for emotional and social support. Even though the diary writers wrote to achieve something in return, their postings also had value for the other readers.

I think they are really brave. I like to read in other peoples’ diaries [laughs]. I can recognize myself [in their writings] and see how other people cope.

Kristin

Some preferred not to write anything on the forum themselves, they accessed the online forum solely to read others’ stories and contributions, and quite a few reported that they learned from reading other patients’ tips and advice. Kristin felt that she had difficulties in expressing herself in writing, but she said that she found great value in recognizing herself in other patients’ stories.

I am not any good at writing myself, so I haven’t. It holds me back. I am not any good at formulating myself. When I read others’ postings, yeah, that is actually how I feel myself. To put things into words is not something I am good at.

Kristin

By reading other peoples’ articulations, we observed that some patients found that their experiences were similar, providing a kind of relief and support because these experiences were seen as being within the *“*scope of normality.” Some patients accessed the online forum to achieve contact with other peers. In some cases, this was articulated directly as presentation rounds, while others were more indirect in their appearances. The possibility of peer communication was more greatly appreciated in some cases than others. Monica, for example, described the fact that her daily life limited her ability to meet others face-to-face.

I think it is alright. I don’t have the physical ability to go out several times a week to meet people. The computer has become my second home [laughs]. Yeah, so I have much contact with others, and my social life is through the computer. Therefore, I have this idea about getting to know people in the same situation.

Monica

The need to come in contact with other patients became evident through the forum observations, and the patients experienced benefits from having access to it, regardless of whether they were active contributors or passive participants.

### Concerns Regarding Self-Disclosure

Observations indicated that some patients were active contributors to the forum, others posted little, and certain patients did not post at all, but followed the discussions. They could therefore be described as lurkers. Linn, who at the time of the interviews was waiting for her operation, expressed that she would very much like to post questions on the forum.

I was really looking forwards to ask about the experiences of the others who are operated. To get some of their experiences, “harvest” of their knowledge, right? That would have been extremely valuable. But then, I think it is really scary to ask the questions, you know?

Linn

She explained that she perceived her literacy abilities as what precluded her from writing on the forum.

I have reading and writing difficulties as well, so when I start writing, it comes out weird. Then, I become even more reserved regarding writing.

Linn

The fear of disclosing her own writing disabilities turned her into a lurker. Observations revealed that a minority of those who had access to the forum were active contributors, and some informants revealed that they often followed the discussion without disclosing their own presence. Some said that they considered their own experiences to be insignificant and therefore did not write anything themselves. However, they reported appreciating reading other peoples’ stories despite the fact that these were without particular highlights or events. Reading these narrations was mentioned as one of the main reasons they accessed the forum. The process of moving from passive participant to active contributor was suggested to be a result of experience. Anne described herself as a *“*forum person*”* because she was an experienced and active participant, but she recalled that she had only become this way through a slow transformation.

I was like that in the beginning. I read a lot before I took the step and started writing myself.

Anne

The fear of disclosing more than one might be comfortable with can be a barrier to actively contributing to such a forum. Hence, Anne’s strategy was to gain confidence by reading forum posts in order to feel eligible to post.

Unlike many online forums, the discussion forum under study did not allow the participants to use nicknames, and the users appeared with their real names when posting. Most said that they did not mind posting to the forum despite the absence of anonymity, but Kristin expressed her view that she would prefer to be anonymous because this would make it easier to introduce sensitive issues and ask difficult questions. Using nicknames provides some degree of anonymity, but there are always certain degrees of self-disclosure related to posting online, as Anne expressed.

It does not bother me. On other forums, even though you don’t have your name, with a nickname, you can find out who the person is anyway. You have to be very careful if you want to be anonymous.

Anne

Even though most did not perceive the lack of anonymity as a personal barrier, some questioned whether this might influence *other* patients’ contributions, as Frank suggested.

For me, it doesn’t matter, because I don’t write anything I don’t want people to know about. So, for my own sake, it doesn’t have any influence, but perhaps, there could be an added feature via which you could post anonymously? There might be those who… not everybody is as open about everything.

Frank

Also, the fact that posting to the forum would reveal that they were part of a bariatric surgery community could be perceived as a barrier to active participation, which is something Kari had thought about–“I think it could be a limitation for others, and many wouldn’t like the fact that other people could know about what they have gone through.” Hence, being open about the surgery is not something everyone likes. Nina mentioned that the fact that only bariatric surgery patients had access made it easier to use the forum. However, she knew several others who had undergone surgery, but preferred to keep it a secret.

I don’t have any problems with it, but others do because I see that among those I have contact with who have had the operation, I know two principals who have undergone surgery. They don’t want to be open and talk about it. They want it to be kept secret…Thus, I think it can be a challenge for some.

Nina

Monica believed that the fact that the forum was moderated meant that it was perceived as more serious than other online health forums, and she held that this prevented people from harassing each other, as she had experienced in other forums–“I believe that when you know that this is more serious, when there are doctors and others (from the clinic) that go through (the postings), then I don’t think people become that childish, letting themselves sink that low…” Forum observations did not identify any form for bullying, harassment, or other negative comments among the participants, and the peer interactions we identified were of a purely supportive character. Everyone has his or her personal limits regarding what he or she is comfortable sharing with others. Because the discussion forum under study was moderated and posting to it did not entail full anonymity, some expressed the feeling that this might increase the participants’ consciousness of what they shared. Kari felt that the demarcation between personal to private sometimes disappeared when people posted online.

I can be personal, but I don’t want to be private…Because there are many things that I think are too private to talk about. People reveal too much. I do not want all that information. Some people need to be protected against themselves. That is just something one has to realize. Some people have no boundaries. You see that on Facebook as well.

Kari

The various degrees of self-disclosure seemed to influence whether the participants felt eligible to actively participate or not. Also, the fact that the forum was moderated appeared to influence how the participants used the forum.

## Discussion

### A Moderated Forum

This study shows that patients who undergo bariatric surgery can obtain information and social support through a moderated online forum and that making such a forum available creates various practices among the patients. The patients were motivated to use the forum by the fact that they must undergo major lifestyle changes that affect both their physical and emotional health. Thus, there is a need for informational and social support. This finding is consistent with previous research that suggests that the desire for both information and social support is a prominent reason for online interaction [26]. The fact that the forum was moderated, and the patients could make contact with health care professionals, meant that the participants experienced the forum under study as being reliable and trustworthy. The participants provided emotional and social support to one another, and we did not identify any communication that was of a disruptive character. This was suggested to be a result of the moderation process, which is in accordance with the findings of Ryan [38].

### The Digital Divide

The digital divide refers to a gap in the access and use of information and communication technology [44,45], and has been a threat to access for poor, minority, and older patients [46-49]. In a recent study that examined underserved patients’ readiness towards patient portal use, Sanders et al found that the majority of the patients did have Internet access and were interested in using a patient portal as a way to manage their care [50]. However, they identified that among those who reported barriers to using the Internet, these were due to interests, know-how, and costs [50]. Because most people have access to computers and the Internet, the challenge of adopting and using these technologies becomes more prevalent, as illustrated in our study. Our findings indicate that some patients experience barriers in participating actively in the forum, implying that there might be a digital divide in this patient population that must be considered when introducing such a solution*.* Sarkar et al did a study on Internet patient portals in diabetes, and concluded that with the health systems increasingly relying on the Internet, those who are at most risk of poor health outcomes might fall further behind, underpinning that the digital divide extends beyond access [49].

### Lurking

The discussion forum served as a source for information and advice, a place for mutual social support and networking with peers. The existence of online forums and communities is dependent on active participation and contributions, but many prefer not to participate publicly [51]. Based on our observations, we found that most were passive participants, who did not reveal their presence in the forum. This behavior can be defined as lurking, which involves seeking answers to questions and viewing and browsing others’ postings, but not actually contributing [51-53]. Participation was uneven in that a minority of the patients contributed to most of the patient-generated content. This is in line with the description of lurkers and posters reported by numerous others [51-54]. There are many reasons for lurking, ranging from the personal to the work-related [51]. In our study, the consideration of self-disclosure, for example, where to draw the boundary between what to share in an online space and what not to, was identified as a factor that restrained active participation. The patients who contributed little or nothing still benefitted from having access to the contributions of other patients because the experiences of these closely resembled their own experiences. This finding is consistent with past studies showing that reading in itself benefit those who lurk in online support groups [30,32,55,56]. Despite their lack of participation, lurkers have the potential for enhanced health promotion through observing or by listening in on others conversations [55]. The fact that the users did not have the opportunity to be anonymous influenced participation. Even though some patients were reluctant to actively participate due to personal barriers, it appears that for others obtaining social support and guidance was of more importance than the issue of self-disclosure. That some patients shared their personal stories shows that the personal benefits of revealing such information are, in some cases, greater than the disadvantages. The fact that patients discussed personal problems online regardless of full anonymity indicates that not being face-to-face with the other participants made it easier to reveal such information. These findings are opposed to those of Kummervold et al, who studied mental health forums in Norway [36]. In their study, the majority of the respondents reported that they would not have participated had they not had the opportunity to use a pseudonym, thus providing full anonymity [36]. However, their respondents also found it easier to discuss personal problems online rather than face-to-face, a finding that is supported by our study [36].

### Study Limitations and Implications

Our study was limited to a qualitative case study, and the findings therefore cannot be generalized. One subject of limitation was the method of recruitment to the interviews, which was done by posting an invitation on the discussion forum. This involved that only those who accessed the discussion forum would see this invitation. One might have achieved contact with other participants if one had used other recruitment methods, such as approaching them by phone. However, this would have involved far more resources than we had available at the time of the study. This study was limited to one discussion forum for bariatric surgery patients, and the results cannot be transferred to other patient populations or other health forums.

There are factors that influence forum participation, thus, determining the degree of engagement and activity. In our study, the mean age of the forum participants was 40 years, indicating that the users were not in the young segment of the population, which uses online communities as an integral part of their daily lives. However, the individuals who obtained access represent a cohort within the population of bariatric surgery patients and therefore provide some implications for future directions. Our findings imply that previous forum experience may influence participation, in that those familiar with online forums may be the individuals who contribute the most. In the literature, a phenomenon called “de-lurking” is described-unfamiliar users begin with reading and getting to know the community to educate and prepare themselves for a more active participation, and eventually write and post themselves [57,58]. It is reasonable to believe that with time, the number of people familiar with such forms of communication will increase and that those who were lurkers in the current study may “de-lurk” with time.

Our findings have some practical implications regarding how such a solution can be used in the context of a bariatric surgery program. First, the patients are unambiguous regarding the value and usefulness of such a moderated discussion forum. Through the forum, the providers have the ability to reach out to those patients that exclude themselves from traditional programs–those who do not show up for traditional consultations, those who experience difficulties in expressing their problems in a face-to-face situation, and those who experience barriers to making contact through conventional methods. Considering the severe outcomes that patients may experience as a result of bariatric surgery, reaching out to those who are in need of informational and social support is crucial. Second, the fact that the users access the forum to read about “new” topics, for example, the weekly topic of relevance, and to read new postings from their peers implies that the forum must be dynamic. This requires the continuous facilitation of the forum, a responsibility of the relevant health care clinic. Third, the fact that the users experience the forum as trustworthy compared to other online forums indicates a great potential for the health care providers to use this channel to deliver the validated health information that the patients need. Thus, one may prevent misinformation and hopefully support the patients’ coping strategies and self-management activities.

In summary, benefits such as social support obtained through interactions with peers and providers motivate patients to actively contribute in an online eHealth forum. However, issues concerning self-disclosure influence whether the patients are comfortable participating actively in the forum or prefer to lurk. Our findings indicate that previous experience with using online forums seems to have an impact because those familiar with the technology may be the individuals who contribute the most. The patients reported benefits from using an online discussion forum, regardless of their active or passive participation, even though active members obtained the greatest advantages in regard to social support and approval.

### Conclusions

The findings of this study imply that a moderated discussion forum for bariatric surgery patients has potential for use in a therapeutic context. The discussion forum fulfills some of the informational and supportive needs of the patients and is particularly useful for those who exclude themselves from traditional programs or experience barriers to making contact with professionals. Even though our findings imply that the patients benefit from using the forum regardless of their active or passive participation, restraining factors, such as considerations regarding self-disclosure, must be further investigated to prevent certain users from being precluded from using such forums in the future.
